# Small-Scale Fluidized Bed Bioreactor for Long-Term Dynamic Culture of 3D Cell Constructs and *in vitro* Testing

**DOI:** 10.3389/fbioe.2020.00895

**Published:** 2020-08-20

**Authors:** Joana Mendonça da Silva, Eloy Erro, Maooz Awan, Sherri-Ann Chalmers, Barry Fuller, Clare Selden

**Affiliations:** ^1^The Liver Group, Institute for Liver and Digestive Health, University College London, London, United Kingdom; ^2^UCL Division of Surgery and Interventional Science, University College London, London, United Kingdom

**Keywords:** fluidized bed bioreactor, scale down model, 3D cell culture, cell encapsulation, hydrogel, alginate

## Abstract

With the increasing interest in three-dimensional (3D) cell constructs that better represent native tissues, comes the need to also invest in devices, i.e., bioreactors, that provide a controlled dynamic environment similar to the perfusion mechanism observed *in vivo*. Here a laboratory-scale fluidized bed bioreactor (sFBB) was designed for hydrogel (i.e., alginate) encapsulated cells to generate a dynamic culture system that produced a homogenous milieu and host substantial biomass for long-term evolution of tissue-like structures and “per cell” performance analysis. The bioreactor design, conceptualized through scale-down empirical similarity rules, was initially validated through computational fluid dynamics analysis for the distributor capacity of homogenously dispersing the flow with an average fluid velocity of 4.596 × 10^–4^ m/s. Experimental tests then demonstrated a consistent fluidization of hydrogel spheres, while maintaining shape and integrity (606.9 ± 99.3 μm diameter and 0.96 shape factor). It also induced mass transfer in and out of the hydrogel at a faster rate than static conditions. Finally, the sFBB sustained culture of alginate encapsulated hepatoblastoma cells for 12 days promoting proliferation into highly viable (>97%) cell spheroids at a high final density of 27.3 ± 0.78 million cells/mL beads. This was reproducible across multiple units set up in parallel and operating simultaneously. The sFBB prototype constitutes a simple and robust tool to generate 3D cell constructs, expandable into a multi-unit setup for simultaneous observations and for future development and biological evaluation of *in vitro* tissue models and their responses to different agents, increasing the complexity and speed of R&D processes.

## Introduction

Three-dimensional (3D) cell culture has gained significant importance by producing physiologically relevant *in vitro* models of *in vivo* processes with complex cell-cell and cell-matrix interactions. However, several current constructs lack vasculature, efficient mass transport and tend to reproduce static or short-term conditions (Li and Cui, 2014; Ramaiahgari et al., 2014; Antoni et al., 2015; Liu et al., 2015). The provision of a dynamic environment *in vitro*, mimicking physiological perfusion, can be generated by bioreactors. They modulate cell performance and phenotype by providing convective mass transfer, overcoming the diffusional limitation of large cell constructs, accurately controlling the local microenvironment and providing mechanical cues and stimuli which lead to cell survival, proliferation and differentiation (Abecasis et al., 2017; Lin and Mequanint, 2017; Yu et al., 2017; Theodoridis et al., 2019). The selection of a bioreactor design is dependent on the 3D cell construct and its inherent physicochemical environment.

A fluidized bed bioreactor (FBB) operates on fluidization principles where a fluid (e.g., culture medium) moving upward through a packed bed of immobilized cells (either on carriers or in capsules) suspends them inducing a fluid-like behavior (Kunii and Levenspiel, 1991). This design benefits from a greater degree of mixing due to the constant circulation of liquid through the solids. It reduces the formation of temperature gradients and promotes homogenous dispersion of the solids (Wang and Zhong, 2007). Consequently, low hydrodynamic shear stress and high heat and mass transfer coefficients are also characteristic of this design (Kunii and Levenspiel, 1991; Werther, 2005). However, for cell culture, FBB requires cell immobilization either on the surface or entrapped within particles, otherwise they would be washed out (elutriated) from the vessel (Wu et al., 2003; Yang, 2003). This provides a 3D structure for cell constructs but may limit the choice of cell type to non-adherent or require specific technology/methods to produce particles which promote adhesion points.

Nonetheless, this design has gained relevance as a technology employed for bioartificial liver devices (BAL), i.e., an extracorporeal machine comprised of a bioreactor with immobilized hepatic cells able to perform liver biochemical functions (Yu et al., 2014; Figaro et al., 2017; Li et al., 2018). For the UCLBAL developed by the UCL Liver Group, >2 L of alginate encapsulated HepG2 cells, initially encapsulated as single cells, are cultured in an FBB for proliferation into several spheroids intra-bead with a tissue-like structure and improved function and performance (Erro et al., 2013; Selden et al., 2013, 2017). In this system, cell spheroids did not displayed necrotic centers and demonstrated increased extracellular matrix production, protein secretion, adaptive stress (e.g., increased expression of antioxidant proteins and decreased protein oxidation) and detoxifying capacity when perfused in liver failure plasma (Selden et al., 2000; Khalil et al., 2001; Coward et al., 2005, 2009; Erro et al., 2013). Once “performance competence” is achieved, the biomass is transferred to the BAL vessel where patient’s plasma would be perfused for treatment.

The scale of a bioreactor is subject to its application and can be classified as micro, benchtop/laboratory, and clinical or industrial scale. Each of these stages can be scaled up or down for different purposes. Increasing the scale is normally associated with an industrial and/or commercial purpose, whereas scaling down from a large size bioreactor creates a model for pre-testing improvements and optimization of process parameters and/or even, other distinctly different applications widening the usage of the system (Li et al., 2006; da Fonseca and Teixeira, 2007).

Although a common method in biotechnology, there is no theoretical model to follow when rescaling a bioreactor. The approaches used comprise fundamental methods; semi-fundamental methods; dimensional analysis and rules of thumb (Garcia-Ochoa and Gomez, 2009). Dimensional analysis and rules of thumb are more practically applied as they are based on similarities between parameters: the up or down scaling of a system is as successful as the number of parameters maintained similar across the different scales (Mandenius, 2016). These are normally main factors influencing or limiting the process including design, geometry, hydrodynamic and kinetic parameters. In a fluidized bed bioreactor, the scale-up or down method identifies similarities in the hydrodynamic performance of the bioreactor and transforms them in dimensionless numbers and relationships, which describe the motion of fluid and solids in a system (Tahmasebpoor et al., 2017). Thus, Glicksman proposed a simplified set of ratios and factors:

(1) $$\frac{\rho_{p}}{\rho }\;\frac{u_{0}^{2}}{\mathrm{gD}}\;\frac{u_{0}}{u_{\mathrm{mf}}}\;\frac{H}{D}\;\phi \;\mathrm{psd}$$

where ρ and ρ_p_ are the density of the fluid and particle, respectively; u_0_ the given superficial velocity; *u*_mf_ the minimum fluidization velocity; g the gravitational constant; H and D the height and diameter of the bioreactor, respectively; ϕ the sphericity of the particle; and psd the particle size distribution (Yang, 2003).

In all circumstances validation of the scaling could be achieved theoretically, utilizing computational fluid dynamics (CFD) tools to calculate fluid velocity and distribution, pressure and shear stress (Villiger et al., 2018); as well as experimentally testing the performance of the bioreactor across scales.

Therefore, based on the technology developed for the UCLBAL, this study aimed to create a novel versatile laboratory-scale fluidized bed bioreactor (sFBB) by scaling-down the clinical scale FBB to a simple, one-piece design. It would constitute a device for long-term culture of micro 3D cell constructs (alginate encapsulated cells), providing a dynamic perfusion environment and hosting significant biomass volumes, which are more similar to *in vivo* than in other small/micro-scale system, while enabling prolonged monitoring and sampling. This bioreactor would move beyond its pilot scale application for the clinical-scale FBB, to a R&D integrated platform for cellular expansion and analysis of the impact of external agents or stimuli on cell behavior and performance, in a “per cell” manner.

## Materials and Methods

### Monolayer Cell Culture

HepG2 cells (ATCC) were cultured in Minimum Essential Medium Alpha Modification (αMEM; GE Healthcare) supplemented with 10% (v/v) fetal bovine serum (Gibco), 100 IU/mL penicillin and 0.1 mg/mL streptomycin (Gibco) and 1.25 μg/ml amphotericin B (Gibco). Cell media was replaced every 48 h, and at approximately 80% confluence cells were harvested or passaged with TrypLE^TM^ Select (Gibco).

### Alginate Hydrogel Encapsulation

The encapsulation procedure has been previously described (Erro et al., 2013). Briefly, harvested cells were mixed 1:1 with 2% (w/v) Na-alginate solution (prepared in 150 mM HEPES-buffered physiological saline; FMC Biopolymer). To reduce buoyancy of produced hydrogel spheres (beads), 1.5% (w/v) density modifiers (glass particles with 2.5 g/cm^3^) were added to the same mix. Hydrogel beads were produced using the JetCutter System (GeniaLab). The equipment was autoclaved and placed in a Class II Biosafety cabinet for aseptic cell encapsulation. A jet from the pressurized stainless-steel vessel containing 300 mL of mix (cell suspension and 2% alginate solution) passed through a 350 μm nozzle positioned above a cutting wire disk and was cut into droplets. These were crosslinked and attained a spherical shape as they fell into a beaker with 204 mM CaCl_2_/155 mM NaCl solution. This produces a 1% (w/v) solid hydrogel without any chemical membrane around the beads. After cross-linking, beads were washed 3× with Dulbecco’s modified Eagle’s medium (DMEM; Gibco) to remove excess calcium. Final volume of produced hydrogel beads was approximately 130 mL per encapsulation run with a cell density of 1.5–1.75 million cells/mL alginate beads, previously optimized (Erro et al., 2013). Encapsulated cell density was validated by nuclei counts performed immediately after encapsulation.

### Scaling Down Methodology

Scaling down methodology followed the empirical similarity rules. The selected design and hydrodynamic parameters of the clinical-scale fluidized bed bioreactor (FBB) (Erro et al., 2013) were the column cross section (A), the height of the settled bed of alginate beads to the diameter of the column (H_b_/D) ratio and the range of superficial velocities (*u*_min_ and *u*_max_, respectively). Estimated values are described in Table 1.

**TABLE 1:** Clinical-scale fluidized bed bioreactor (FBB) design and hydrodynamic parameters for developing a scale-down prototype.

**FBB parameters**	
A (m^2^)	0.0177
u_min_ (m/s)	0.00021
u_max_ (m/s)	0.00040
*H*_b_ (cm)	14.15
*H*_b_/D	0.94
*H*_t_/D	2.33

*Cross section (A), minimum and maximum superficial velocities (*u*_min_ and *u*_max_, respectively), height of the settled bed of beads (*H*_*b*_), height (*H*_*t*_), and diameter (D) of the column.*

### Small-Scale Fluidized Bed Bioreactor

**FIGURE 1:**
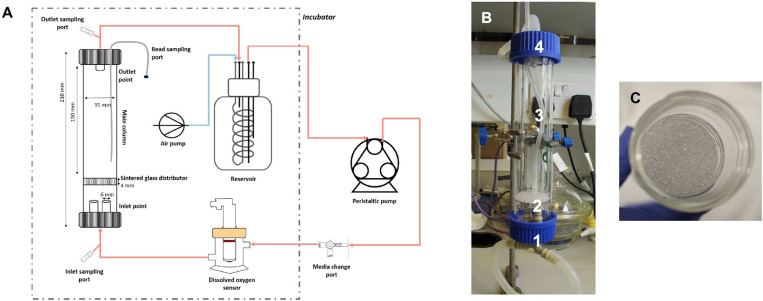
Small-scale fluidized bed bioreactor. **(A)** Schematic representation the system setup. Culture media is perfused through the sFBB from the inlet to the outlet and recirculated to reservoir by the peristaltic pump. The setup operates inside a 5% CO_2_ incubator (dotted box) except for the pump. Active gassing is achieved by gassing the reservoir through a silicone coil placed inside and connected to an air pump. Media changes are performed through the media change port on days 4, 7, 9, and 11. **(B)** sFBB prototype: 1 – inlet tubes; 2 – sintered glass distributor, with structure highlighted in **(C)**; 3 – glass column; 4 – outlet tubes.

The small-scale fluidized bed bioreactor (sFBB) comprised of a 21 cm long and 3.5 cm diameter glass column fitted with 4 mm thick sintered glass distributor, placed 2 cm from the bottom. It was sealed with two GL45 thread safety caps: bottom cap included two vertical inlet points, while the top included an outlet and a sampling port to retrieve beads. This bioreactor was connected in a closed loop to a reservoir through silicone tubing (Altec) and recirculation of liquid achieved using a peristaltic pump (Watson-Marlow) at flow rates chosen to maintain a fluidized bed of ∼1.6 to 2-fold. Media changes were carried out through the media change port, pumping out the spent media and in the fresh supply. Inside the reservoir, a coil of gas permeable silicone tubing was fitted for active gassing and connected to an air pump or an oxygen concentrator (AirSep), with the flow regulated by a flowmeter. The main components of the system were reusable, washable and autoclaved for 20 min at 121°C. Figure 1 provides a schematic of the setup of the system.

### Flow Simulation

A model of the sFBB was produced using computer-aided design (CAD) software (SolidWorks^®^ v.2018, Dassault Systems). Theoretical analysis of the bioreactor hydrodynamic performance was carried out by computational fluid dynamics (CFD), using a flow simulation package (SolidWorks^®^), in which the numerical method followed the finite volume element model. Parameters and approximations of the system are described in Tables 2, 3, with the sintered glass distributor defined as porous media.

**TABLE 2:** Initial conditions set for CFD simulation.

**Initial conditions**	
Temperature (°C)	37
Pressure (Pa)	101325
Gravitational constant (m/s^2^)	9.81
Fluid	Water
Intel flow rate (mL/min)	12–25
Flow type	Laminar and Turbulent
Wall thermal condition	Adiabatic
Boundary conditions	Inlet at uniform flow rate
	Outlet at environmental pressure
Mesh	Global mesh refinement: 3
	Local mesh refinement: 4

**TABLE 3:** Porous media conditions defined in CFD database.

**Porous media**	
Porosity	0.5
Permeability type	Unidirectional
Resistance calculation formula	*k* = μ/(ρ.D^2^)
Pore size (m)	0.000375

### sFBB Equilibration Time

30 mL of alginate beads were added to the sFBB and the reservoir filled with 500 mL of 2 mM CaCl_2_/155 mM NaCl solution. Flow rate, set by a pre-calibrated peristaltic pump, fluidized the bed of beads to the intended expansion level, from 1.2 to 2-fold. Height of the expanded bed was measured in millimeters through the glass. 0.3 mL of 5 mg/mL of methylene blue solution (ProVepharm) was injected at the inlet fluid sampling port and a time course of the sFBB mixing profile was established by collecting fluid samples from the outlet sampling port at fixed time points. The absorbance of each sample was measured in triplicate at 666 nm.

### Bed Fluidization in the sFBB

30 mL of alginate encapsulated HepG2 cells were collected and washed 3× with 2 mM CaCl_2_ solution. 10 mL were stained for 10 min with 1 mg/mL toluidine blue solution (Sigma), washed thoroughly and drained to remove excess dye. They were added to the sFBB and topped with 20 mL of non-stained beads. The reservoir was filled with 500 mL of 2 mM CaCl_2_ solution and flow rate set to expand the bed to 1.6-fold its initial height (measured in millimeters through the glass). A video of fluidization was recorded to identify the instants of expansion and homogeneity.

### Mass Transfer in the sFBB

Mass transfer in the system was determined using 0.03 mg/mL FITC-dextran 150 kDa solution (prepared in 2 mM CaCl_2_; Sigma). To measure uptake under static conditions, 100 μL of dextran solution was added to 10 μL of empty alginate beads (i.e., not containing cells) per well in a 96-well black microplate and imaged diffusion immediately using Nikon Eclipse Ti-E microscope with Hamamatsu Flash 4.0 sCMOS camera and Nikon C2 Confocal with PMTs for three channel simultaneous imaging. Mass transfer out of the beads, required overnight incubation in dextran solution, replaced by 100 μL of 2 mM CaCl_2_ solution immediately before imaging.

For transfer within the sFBB, with a bed fluidizing 1.6-fold its initial height (flow rate at 18 mL/min), 0.6 mL of 10 mg/mL FITC-dextran solution was injected for a final concentration of 0.03 mg/mL in the system. Beads were then sampled at fixed time points, 10 μL dispensed per well and imaged. To study transfer out, the same procedure was followed, after adding fresh 2 mM CaCl_2_ solution to the reservoir. Inherent to the experimental setup, between sampling and imaging there was a 3-min interval.

### Culture of Alginate Encapsulated HepG2 Cells

After encapsulation of HepG2 cells, alginate beads were cultured in the sFBB in αMEM supplemented with 10% (v/v) human plasma, 100 IU/mL penicillin and 0.1 mg/mL streptomycin (Gibco) and 1.25 μg/ml amphotericin B (Gibco) for 12 days at a bead to media ratio of 1:46. Running inside a 5% CO2 dry incubator (LEEC) at 37°C, fluidization at 1.6-fold was maintained constant by adjusting the flow rate throughout culture, ranging from 9 to 23 mL/min, depending on cell density. The media change was carried out as described in section “Small-Scale Fluidized Bed Bioreactor” with the following regime: 50% on day 4 of culture, 60% on day 7, 70% on day 9, and 80% on day 11. Similarly, encapsulated HepG2 cells were cultured in conventional tissue culture flasks at the same bead:media ratio, and also in the scale-up fluidized bed bioreactor (FBB) as detailed previously (Selden et al., 2017).

### Cell Proliferation of Encapsulated Cells

Encapsulated cells in alginate beads were washed with HBSS (Gibco). To release cell spheroids from the hydrogel, 4 mL of 16 mM EDTA solution (pH 7.4) were added to 0.3 mL of beads. Cells were pelleted, resuspended in PBS (Gibco) and disaggregated with 21-gage needle into single cells. They were then lysed and stabilized by mixing in the lysis and stabilization buffers, according to the manufacturer’s protocol (Chemometec). Nuclei in suspension were quantified using NucleoCassette^TM^ in Nucleocounter NC-100^TM^.

### Cell Viability of Encapsulated Cells

Viability of alginate encapsulated HepG2 cells was assessed through live/dead assay using Fluorescein diacetate (FDA)/Propidium iodide (PI) staining. Alginate encapsulated cells (approximately 0.25 mL) were washed twice with PBS and re-suspended in 0.5 mL of PBS. Next, beads were stained with 20 μl PI and 10 μl FDA for 90 s, washed again with PBS and transferred to a microscope slide. Five different images were captured (for a total of 100 beads imaged) using Nikon TE200 microscope and cell viability calculated based on fluorescence intensity. This method has been thoroughly validated and described in detail in (Selden et al., 2017).

### Alginate Bead Dimension

Phase contrast images of alginate HepG2 cell beads were taken with a Nikon TE200 microscope equipped with a Nikon DS-Fi1c camera and DS-U2 PC control unit. The NIS-Element Microscope Imaging software was used to measure the diameter and aspect ratio of the imaged beads.

### Statistical Analyses

Statistical analysis was carried out with GraphPad Prism software. To compare two groups, Student’s *t*-test corrected for the Holm-Sidak method was applied. When more than one group was compared, analysis of variance (one-way ANOVA) was carried out, corrected for Tukey’s method. Significance levels were set at *p* < 0.05 and details of sample numbers, replicates, data and error description are described in each figure legend.

## Results

### Scaling-Down of Fluidized Bed Bioreactor Through Empirical Similarity Rules

For the scale down of the FBB, dimensional analysis was applied selecting important design and hydrodynamic parameters of a fluidized bed, since the biological components of the system (i.e., cell type, culture media and hydrogel cell beads properties) would be the maintained across scales. Using encapsulated HepG2 cells enabled to directly validate the model with the clinical scale system. The settled bed height to diameter of the bioreactor ratio (H_b_/D) influences the fluid velocity and consequently the mixing in the bioreactor, an in turn, the fluid superficial velocity correlates directly with the minimum fluidization velocity and subsequent expansion levels.

Thus, maintaining the two parameters (H_b_/D ratio and the fluid superficial velocity) (Table 1), and establishing the minimum volume of beads hosted by the sFBB prototype to be 30 mL, comparable to a small animal liver, resulted in an iterated minimum diameter for the small-scale bioreactor of 3.5 cm. The minimum height for the column was 8.2 cm given by the H_t_/D ratio, just enough to sustain the expansion of the bed to twice its settled height. However, to increase the bioreactor flexibility to host larger volumes of alginate beads and expand the bed to more than double its settled height, while still guaranteeing a safe distance from the outlet to prevent beads escaping (elutriation), the height was extended to 15 cm.

### sFBB Computational Fluid Dynamics Analysis

As a theoretical pre-evaluation of the prototype hydrodynamic performance and to validate the scale-down model and construction elements of the design, CFD modeling of the sFBB determined the fluid velocity, trajectory, pressure and shear stress. Of note, the simulated models only considered fluid behavior and did not include the solids phase, since the aim was to validate the design and distributor.

**FIGURE 2:**
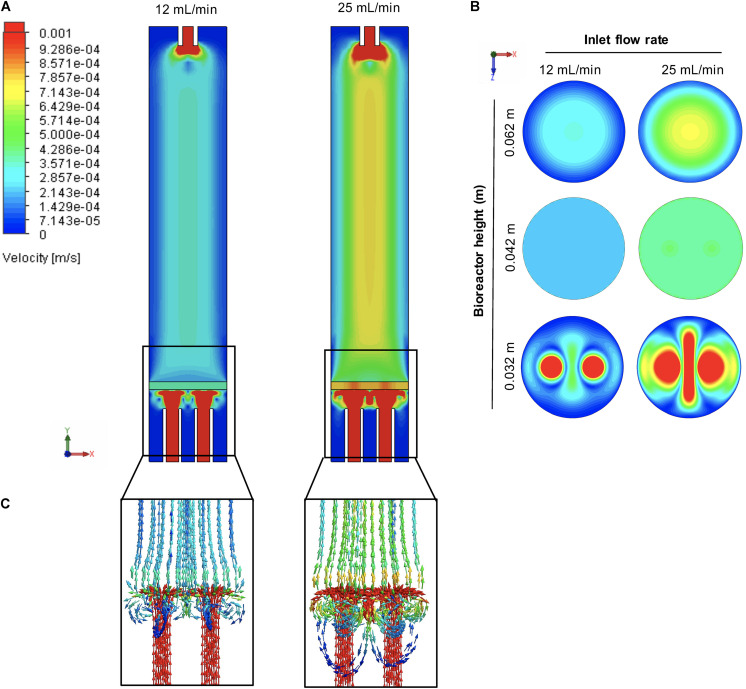
Flow regime inside the small-scale fluidized bed bioreactor (sFBB). **(A)** Velocity cut plots of the middle plane of sFBB at inlet flow rates of 12 and 25 mL/min, as well as **(B)** top plane plots at 0.032, 0.042, and 0.062 m from the base of the bioreactor. Fluid trajectory of is also depicted in **(C)**, focusing on the inlet-distributor region. Values presented in m/s.

Two distinct inlet flow rates were analyzed: 12 and 25 mL/min, corresponding to the fluid superficial velocity range maintained for the scaling down. Velocity profiles corroborated that faster velocities were obtained at higher inlet flow rates (Figure 2). On average, velocities attained in the system were 2.095 × 10^–4^ m/s (min = 3.75 × 10^–9^ m/s and max = 0.021 m/s), and 4.596 × 10^–4^ m/s (min = 2.14 × 10^–8^ m/s and max = 0.043 m/s) for 12 and 25 mL/min, respectively. These results verified the validity of the scale down-model as the simulated values fitted the interval of intended superficial velocities. Moreover, the highest velocities were achieved at the inlet and outlet points (Figures 2A,B, 0.032 m distance) and the flow was almost homogenously dispersed immediately above the distributor (Figure 2B, 0.042 m distance). As the fluid moved through the column, the wall effects became more prominent, with the resultant color gradient indicating the formation of a stagnant fluid layer near the wall in contrast to the faster flows observed in the center (dark blue layer; Figure 2B, 0.062 m distance; Supplementary Figure 1).

Flow trajectory plots indicated the flow was axially oriented (along *Y*-axis), and radial flow (*XZ*-axis) primarily occurred in the calming zone between the surface of the inlet point and the distributor (Figure 2C), suggesting the flow was predominantly laminar, possibly more turbulent below the distributor.

**FIGURE 3:**
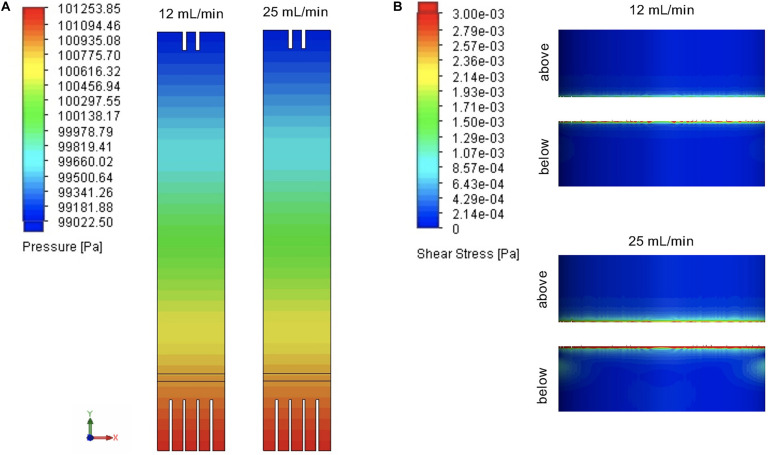
Fluid pressure and shear stress profiles in the small-scale fluidized bed bioreactor (sFBB). **(A)** Pressure cut plots of the middle plane of sFBB at inlet and **(B)** flow shear stress surface plots on the inner wall of the bioreactor focused on the distributor region (below and above the distributor). Simulations were computed for inlet flow rates of 12 and 25 mL/min. Values are presented in Pa.

This system displayed a progressive pressure loss across the column with the inlet at 101253 Pa and the outlet at 99022 Pa (Figure 3A), although still within the range of atmospheric pressure. The pressure drop across the distributor was 38.5 Pa. Results also demonstrated that pressure was not a function of the inlet flow rate as the two profiles were identical. Whilst shear stress in the sFBB was generally low, higher values were noted in regions immediately below and above the distributor (Figure 3B), and at inlet and outlet points correlating with the faster fluid velocities. Nonetheless, numerically, shear stress values did not exceed 0.23 Pa (at the outlet).

Overall, CFD analysis verified that the sFBB prototype would effectively and homogenously disperse the fluid, verifying the initial pre-requisite of the performance of the design.

### sFBB Bed Fluidization and Expansion

**FIGURE 4:**
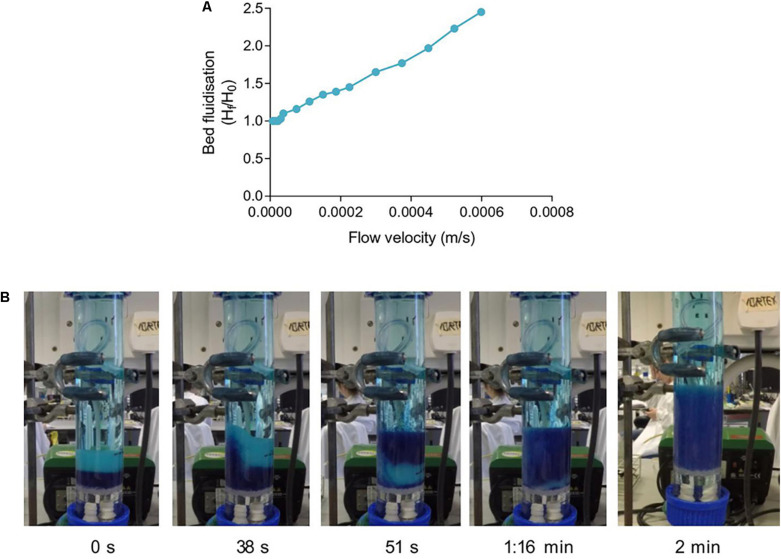
Fluidization behavior in the small-scale fluidized bed bioreactor (sFBB). **(A)** Expansion level of a bed of alginate encapsulated cells in the sFBB as a function of increasing superficial velocities. **(B)** Time lapse and fluidization pattern of a 2-fold bed expansion homogenously achieved after 2 min.

After theoretical evaluation, fluidization efficacy of the sFBB prototype was experimentally validated through bed expansion level and pattern. Increases in the flow rate determined that expansion of the bed (1.03-fold) firstly occurred at a superficial velocity of 3.00 × 10^–5^ m/s constituting the experimental minimum fluidization velocity (*u*_mf_) of the sFBB (Figure 4A), which was not in agreement with theoretical predicted values (Supplementary Table S1). Bed fluidization followed a linear correlation with increasing fluid superficial velocity, expanding proportionally. A 1.6-fold expansion was attained at 2.69 × 10^–4^ m/s and a 2-fold at 4.40 × 10^–4^ m/s.

Observations of the bed fluidization pattern determined that the upward movement of the fluid, as it entered the sFBB, induced the hydrogel beads to mix in a bottom-top pattern, transferring a considerable portion of the bottom layers (blue stained beads) to the top of the unstained beads. Although in an initial heterogeneous fashion as noted by some low-velocity spots at the bottom of the bed (1:16 min) (Figure 4B), a 2-fold homogenous bed expansion (from 2.6 to 5.3 cm) was achieved after 2 min of fluid recirculation. Continuous observation identified a steadily fluidized bed, maintaining the expansion level, with beads moving downward near the wall and upward in the center of the column.

These results demonstrated the system was able to induce and sustain a stable and coherent fluidization of a hydrogel bed.

### sFBB Equilibration Time

**FIGURE 5:**
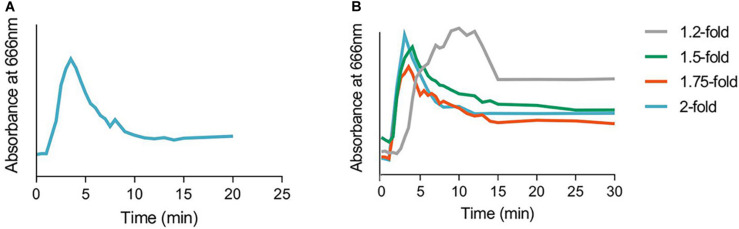
Mixing pattern in the small-scale fluidized bed bioreactor (sFBB). **(A)** Mixing profile and times in the sFBB at 1.6-fold bed expansion after injection of 0.3 mL of methylene blue (5 mg/mL) and **(B)** the variation at different expansion levels (from 1.2 to 2-fold the settle height). Absorbances of retrieved samples measured at 666 nm.

Mixing time of a bioreactor is an important parameter to determine the capacity of the system to reach equilibrium and homogeneity. Upon injection of methylene blue at the inlet sampling port in the sFBB fluidizing at 1.6-fold, time course analysis detected the first increase in absorbance (at the outlet sampling port) after 2 min and, the maximum at 3.5 min constituting the circulation time of the sFBB, i.e., the time particles take to flow through the bioreactor (Figure 5A). As the absorbance decreased, a second peak was observed at 8 min, defining the recirculation time of the system as 4.5 min. The profile stabilized at 11 min of operation with the system returning to equilibrium after the disturbance (dye injection) was introduced. Thus, the mixing time (*t*_m_) of the bioreactor was 11 min.

The mixing conditions in a fluidized bed were a function of the flow rates, which consequently impacted the level of fluidization. For a minimum 1.2-fold bed expansion (at 1.15 × 10^–4^ m/s), color was first detected at 3 min, but the maximum was only achieved at 10 min and equilibrium after 15 min (Figure 5B). As the bed progressively expanded to 1.5, 1.75, and 2-fold its settled height, maximum absorbance was attained at 4, 3.5 and 3 min, respectively, and the equilibrium at 13, 11, and 12 min. This demonstrated a correlation between mixing time and fluidization level, with low flow rates increasing the equilibration time of the system. At the desired operating conditions, i.e., a bed fluidizing at 1.6 to 2-fold, the system attained equilibrium at 11 to 12 min.

The theoretical mixing time of the a fluidized bed bioreactor with liquid recirculation approaches the completely mixed flow (CMF) (or continuous stirred tank reactor - CSTR) model more than the plug flow mixing (Andrews, 1988; Gòdia and Solà, 1995). According to the former, the hydraulic retention time, i.e., the length of time a particle remains in the system, is given by:

(2) $$\theta =\frac{V}{Q}$$

where V is the volume and Q the volumetric flow rate. This is the characteristic time of the system (θ = τ) and in the case of recirculation, the recirculation time of the system. The mixing time (*t*_m_) correlates with τ, assuming 95% of complete mixing, as:

(3) $$t_{m,95\%}=3\,\tau$$

In the sFBB system, considering channeling effect due to the stagnant volume of the reservoir (Fogler, 2006) and the recirculating volume corresponding to 47% (32% in the sFBB and 15% in the tubing) of the total 500 mL, at a flow rate of 25.7 mL/min, θ = 9.1 min. From Eq. (2), the theoretical mixing time of the system resulted in 27.2 min.

The theoretic θ and *t*_m_ did not agree with the experimental 4.5 and 11 min, respectively. However, experimental *t*_m_ could be approximately described by Eq. (2):

(4) 3×4.5=13.5⁢m⁢i⁢n

with a deviation of 1.5 min from the interval of 11–12 min.

### sFBB Mass Transfer

To determine mass transfer efficiency in the bioreactor, relative fluorescence intensity (intensity/area of the region of interest) of fluorescein isothiocyanate-labeled 150 kDa dextran (FITC-dextran) was quantified in both alginate bead and media milieu.

**FIGURE 6:**
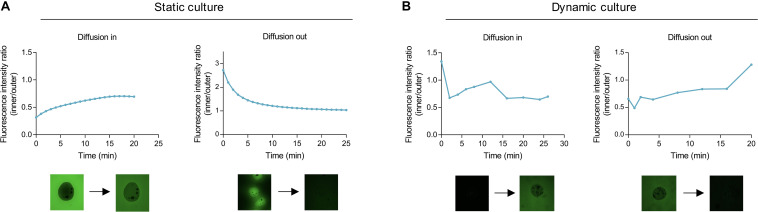
Mass transfer in alginate beads under static and dynamic cultures. Representative diffusion of FITC-labeled 150 kDa dextran from the surroundings into alginate empty beads (not containing cells) and vice-versa in static **(A)** and dynamic **(B)** conditions. Values presented as ratio of relative fluorescence intensity (RFI) inside to RFI outside of the bead.

When exposed to FITC-dextran solution in static conditions, transfer of particles into the alginate beads was slow, plateauing at 18 min with the inner to outer relative fluorescence ratio not reaching 1 during the monitored time (i.e., relative fluorescence not equalized between the two phases) (Figure 6A). Conversely, dextran transfer out of the bead occurred rapidly during the first 10 min, slowing thereafter and attaining 1 after 20 min.

Under hydrodynamic conditions, it was observed that transfer into the beads reached a ratio of 1 after 12 min, diminishing and stabilizing afterward as a consequence of fluid recirculation (Figure 6B). In comparison with static culture, where, after 20 min, the unit ratio was not attained, the dynamic environment enabled faster mass transfer into the alginate hydrogel. Transfer out continued to occur at a fast rate with relative fluorescence equalizing at 17 min. Hence, the sFBB effectively promoted mass transfer in the alginate beads, faster than in static and with the alginate hydrogel not constituting a physical barrier for particle size up to 150 kDa.

### Biological Performance of sFBB

**FIGURE 7:**
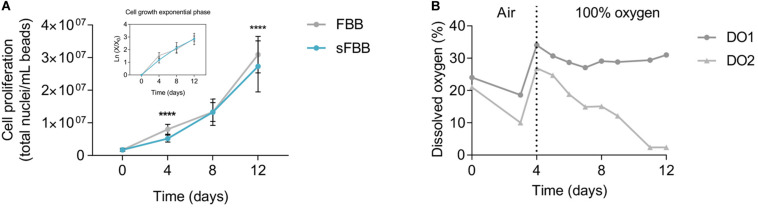
Biological performance of the small-scale fluidized bed bioreactor (sFBB). **(A)** Cell proliferation throughout 12 days of culture analyzed for the sFBB and compared to the clinical scale FBB, with representation of the exponential growth phase. Data presented are average ± standard deviation (SD), and average ± standard error mean (SEM) for the exponential growth phase (*n* = 17 for FBB and *n* = 7 for sFBB). Statistical analysis assessed by multiple Student’s *t*-test. *p < 0.05, ***p < 0.001, ****p < 0.0001. **(B)** Representative oxygen supply and consumption in the sFBB during the 12 days of culture with dissolved oxygen (DO) monitored at the inlet (DO1) and outlet (DO2) points. Bioreactor operated in a controlled environment of 5% CO_2_ during the 12 days, with active gassing up to day 3 of the same gas mix and replaced by pure oxygen for the remaining days.

**FIGURE 8:**
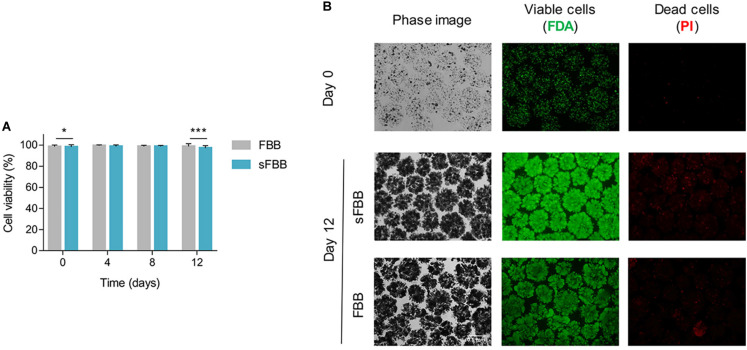
Cell viability in the small-scale fluidized bed bioreactor (sFBB). **(A)** Cell viabilities of alginate encapsulated HepG2 cells throughout 12 days of culture analyzed for the sFBB and compared to the clinical scale FBB. Data presented are average ± standard deviation (SD) (*n* = 17 for FBB and *n* = 7 for sFBB). Statistical analysis assessed by multiple Student’s *t*-test. ***p < 0.001. **(B)** Cell bead morphology (phase image) and viability (live cells – FDA, dead cells – PI) images at 10× magnification from days 0 and 12.

Final validation of the prototype was its biological efficacy, determining its ability to host and expand viable biomass to a similar level to the clinical-scale FBB. Alginate encapsulated HepG2 cells cultured for 12 days in the sFBB proliferated into several multicellular spheroids with a final density of 27.3 ± 0.78 million cells/mL beads (*n* = 7). The growth curve in the sFBB was similar to that in FBB, although the later yielded a significantly higher density of 30.9 ± 0.56 million cells/mL beads (*p* < 0.0001) (Figure 7A). In both systems, for the time frame between 4 and 12 days, cell growth was in the exponential phase. Cell viability was maintained above 97% throughout the experiment in both systems with a difference of 1.5% between them on day 12 (Figure 8). These small variations could be attributed to the design modifications (particularly the distributor) but considered negligible as values fell within the desired range of cell density and viability.

Monitoring the dissolved oxygen (DO) levels, at the inlet (DO1) and outlet (DO2) points of the sFBB, further demonstrated that cells were metabolically active throughout the 12 days as suggested by a progressive decline in DO2, i.e., as cells proliferated, oxygen consumption was increased and the concentration of oxygen in media declined (Figure 7B).

Of note, no contamination was detected in the sFBB during culture period, proving the ability of the prototype to sustain closed culture conditions in a non-sterile environment (i.e., inside an incubator).

These results corroborated the effective scale down of the FBB, with the prototype (sFBB) supporting 12 days of continuous culture of 3D cell constructs, maintaining them highly viable and promoting proliferation to cell densities equivalent to the clinical scale bioreactor.

#### Alginate Bead Integrity

**FIGURE 9:**
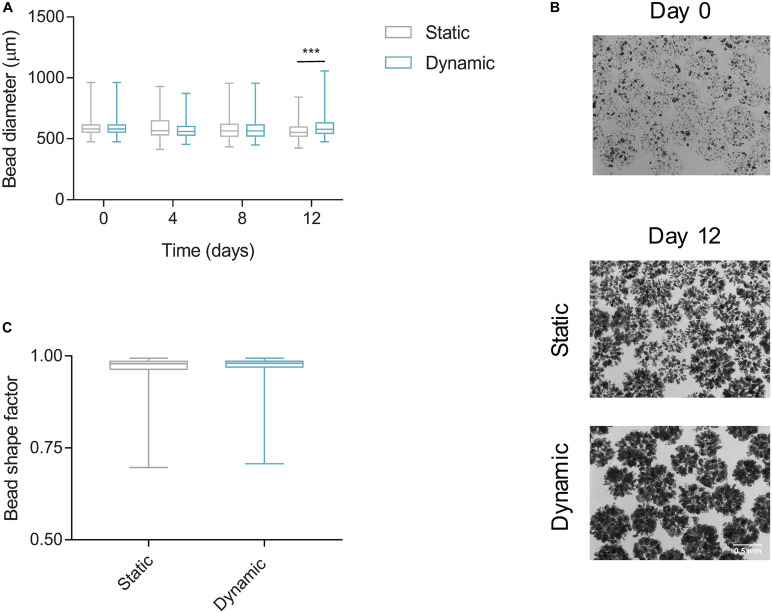
Alginate bead integrity and diameter in the small-scale fluidized bed bioreactor (sFBB). **(A)** Diameter of alginate beads encapsulating HepG2 cell spheroids cultured under static and dynamic (sFBB) conditions for 12 days. **(B)** Aspect ratio of beads on day 12 in static and dynamic culture. **(C)** Phase images captured on day 0 and 12. Data presented in box plot are average with whiskers representing minimum and maximum values (*n* = 4). Statistical analysis assessed by multiple Student’s *t*-test. ***p < 0.001.

The integrity of the micro hydrogels fluidized in the small-scale prototype had to be preserved to support the encapsulated cells and their long-term culture. During the 12 days of constant fluidization, the diameter of the alginate beads did not alter significantly, except on day 12 where the average bead diameter was 606.9 ± 99.3 vs 573.6 ± 77.8 μm in static conditions (*p* < 0.001) (Figure 9A). This increase was likely an outcome of cell growth since spheroids formed under dynamic conditions were denser and larger than in static (Figure 9B). The integrity of the beads was further evidenced by the preservation of their spherical shape and 0.96 shape factor on day 12 (Figure 9C). Thus, the constant flow inside the bioreactor did not produce sufficient shear stress to cause wear and/or disintegration of alginate beads and corroborated the low shear stress values simulated by CFD analysis.

Of note, the sustained diameter, and subsequent volume, of the alginate beads throughout the initial culture days indicated that bead swelling was not a significant factor impacting the hydrodynamic behavior of bed nor the accuracy of theoretical calculations such as *u*_mf_.

#### Multi-Unit Setup for Simultaneous Observations

With the performance of the prototype validated, the possibility of expanding it into a setup of multiple parallel units was explored, as it would be particularly beneficial to investigate several conditions simultaneously in a dynamic environment, or to use the device as a co-culture system. The setup was incrementally expanded up to four sFBBs and the main challenge was providing the same hydrodynamic conditions to each bioreactor. It was observed that branching the flow after the pump to feed more than one bioreactor created pressure inconsistencies which resulted in deficient fluidization, with beds fluidizing at different levels or even not fluidizing at all (data not shown). Fitting a multichannel head in a peristaltic pump provided the ideal solution to enable individual circuits for each bioreactor, allowing them to run in the same setup without increasing the amount of associated equipment (i.e., pumps) or resulting in lagged operation between columns.

**FIGURE 10:**
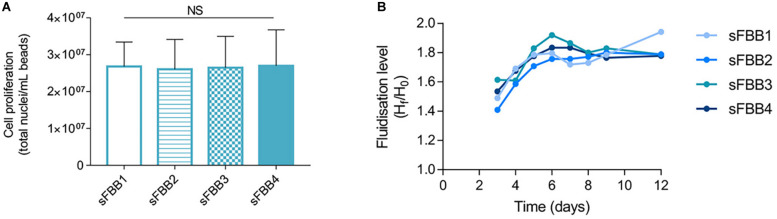
Performance of multiple sFBB setup. **(A)** Cell density on day 12 of culture in four sFBBs (sFBB1, sFBB2, sFBB3, and sFBB4) operating in parallel. Data presented as average ± SD (*n* = 3). Statistical analysis assessed by one-way ANOVA with Tukey’s correction. NS – non-significant. **(B)** Representative fluidization levels throughout culture of four sFBBs in parallel.

Biological assessment demonstrated that final cellular densities (day 12) were not significantly different across all four bioreactors (Figure 10A) and were comparable to the final yield of a single unit setup (Figure 7A). Furthermore, in each bioreactor, bed expansion followed a similar pattern to sustain a fluidization level of at least 1.6-fold (Figure 10B). The flow rate was adjusted throughout the 12 days to account for spatial reorganization of the bed and weight of the beads due to cell growth.

## Discussion

Although fluidized bed bioreactors could conceptually create a dynamic environment for 3D cell constructs that more closely replicates the *in vivo* perfusion, this design has not been widely explored for *in vitro* models in Tissue Engineering. Therefore, the aim of this study was to develop a laboratory-scale FBB (sFBB) that could be a simple and versatile device for long-term culture and analysis of hydrogel encapsulated cells providing both the 3D structure and flow perfusion.

Using established FBB technology applied to the clinical scale UCLBAL (Erro et al., 2013), a scale-down model based on empirical similarity rules was developed, focused on design and hydrodynamic parameters. This approach proved to be simple and effective in creating a benchtop model with identical biological performance. Dimensional analysis has the advantage of reducing the number of variables in a problem and simplifying their estimation compared to fundamental methods, which resort to complex equations (Garcia-Ochoa and Gomez, 2009). However, this method requires prior knowledge of the behavior of the system and the independent variables that impact it the most (Albrecht et al., 2013). In a fluidized bed even if fluid superficial velocity, solids density and their circulation pattern are maintained, the hydrodynamics of the bed might not be the same. Therefore, since the biological components were the same between scales, selecting a design parameter such as H_b_/D ratio to completement those similarities will highly contribute to sustain the intended fluidization level with the required interphase mixing (Bello et al., 2017). This analysis only took into consideration physical and geometric correlations, not including parameters associated to the biological performance which will be fundamental for bioprocesses highly sensitive to minor fluctuations in the culture composition (e.g., glucose concentration, oxygen tension). It would, however, require detailed experiments to determine the variables of the limiting steps of cellular growth/performance, while the geometric analysis provides a more straightforward approach.

The sFBB design was conceptualized as a one-piece central column fitted with a sintered glass flow distributor. Some systems do not include a flow distributor, reasoning on channeling effects created by this feature (i.e., preferential circulation of fluid through specific arteries generating points of fast moving particles interspersed with stagnant ones), and that without it the bioreactor still performs accordingly (Agu and Moldestad, 2018). However, the majority describes the distributor and its design as the key parameter for the performance of the FBB. Lu et at developed a distributor based on a turbine mechanism capable of fluidizing the bed horizontally, exposing all microcapsules to the same level of perfusion and minimizing wear (Lu et al., 2016). Uniformity of the flow is not the only criteria for a better performance, Wormsbecker et al., demonstrated that the punched plate distributor promoted a faster drying of solids than a perforated plate due to generated lateral mixing (Wormsbecker et al., 2007). According to the computational flow simulation data and experimental observations, the flow out of the vertical inlets was axially oriented by the distributor and evenly dispersed throughout its surface area corroborating the purpose of this feature in the homogenization of the flow. Its performance was further validated with the uniform expansion of the bed, demonstrating that the homogeneous dispersion of the flow constituted a pre-indication of the fluidizing performance of the bioreactor (Zheng and Zhu, 2003; Sobrino et al., 2009; Sun et al., 2017). Nonetheless, the influence of the distributor is not only intrinsic to its design but subjected to the properties of the solids: for example, its effect is greater in beds of smaller particles (Rahimpour et al., 2017).

Fluidization of alginate beads started at 3.00 × 10^–5^ m/s and was linearly correlated to the increasing fluid superficial velocity. Although the linear trend is in accordance with literature, theoretical prediction of the minimum fluidization velocity differed from the experimental results (Supplementary Table S1) (Ergun, 1952; Wen and Yu, 1966; Yang, 2003; Huang et al., 2018). Studies have highlighted these discrepancies attributing them, for example to the third order dependence on the bed porosity in Ergun’s equation, a parameter difficult to accurately determine experimentally, or the semi-empirical correlations being established for the particular conditions of the model they describe, which are not universal to all fluidization systems (e.g., solid size, bed porosity, flow regime) (Asif, 2010; Sulaymon et al., 2013; Kramer et al., 2019). Yet, expansion to twice the initial settled height was achieved within 2 min and followed patterns consistent with others described, where solids move upward in the center of the column and downward near the walls due to the drag (Fede et al., 2013). Similarly, Legallais et al. observed the bottom-top mixing pattern and stabilization of the expanded bed in under 5 min, a process which was independent of the volume of beads and perfusion flow (Legallais et al., 2000).

The flow perfusion that effectively fluidized the hydrogel bead bed also achieved an average circulation time of 3.5 min and a mixing time of 11–12 min (Figure 5), which were comparable to those reported in other studies, verifying the compliance of the system in following a recirculation pattern (Pedersen et al., 2016). It also demonstrated the efficacy of the bioreactor in resolving disturbances and minimizing concentration gradients in the liquid milieu (Cabaret et al., 2007; Rodriguez et al., 2014; Zivkovic et al., 2017). Although the mixing time could be approximately described by the CMF model (*t*_m_ = 3τ), the experimental τ did not agree with the theoretical time. The overestimation could result from the model not taking into consideration a possible channeling effect generated by the faster fluid velocities in the center of the bioreactor (Supplementary Figure 1). If the bulk of the fluid travels at the faster velocities, then it governs recirculation, subsequently reducing τ.

Under these dynamic conditions, mass transfer into the alginate beads occurred approximately in 12 min, whereas in static environment it could not be determined. Although testing of the dynamic environment included an interval between sampling and imaging of beads, because the transfer in static conditions was much slower than in dynamic, as seen by the significant differences in transfer times, this delay did not distort the analysis. Therefore, the premise that mass transfer is faster in dynamic cell culture was confirmed (Salehi-Nik et al., 2013). Transfer times were similar to the equilibrium time of the bioreactor suggesting the governing mechanism in the system was the convection movement of the fluid, transporting solutes quicker to the outer surface of the alginate beads and subsequently, influencing the diffusion within the hydrogel which depends on the concentration gradient at the bead-fluid boundary.

Mass transfer times and mechanism were identical to those described for molecules such as vitamin B12 or albumin (smaller than 150 kDa) in 2.2% (w/v) alginate beads (David et al., 2004). These rates are a function of the diameter, composition and mechanical properties of the hydrogel, as well as, of the size of the diffusing particles and thus, smaller molecules could diffuse at the same rate as larger ones for hydrogels of different compositions (Gautier et al., 2011; Sun et al., 2018). Of note, the physical barrier imposed by the hydrogel did not impair mass transfer of 150 kDa dextran, reinforced by studies demonstrating alginate permeability to large molecules up to 500 kDa (Khalil et al., 2001; Mobed-Miremadi et al., 2014). Design parameters of fluidized bed bioreactors can also influence mass transfer: increasing the number of holes and pressure drop across the distributor increases the mass transfer coefficient (Kalaga et al., 2014).

The physical performance of the sFBB was corroborated with its biological outcome. It yielded comparable viable cell numbers to the clinical scale FBB as viability and cell density were within the intended interval. Minor differences between scales have also been reported in other scale-down models, for example in viable cell number or metabolic activity either due to uncontrolled parts of the process or just a consequence of inherent biomass variability (Zeilinger et al., 2011; Vodopivec et al., 2019). Furthermore, hydrogel beads maintaining their integrity and sphericity throughout 12 days (and equivalent to those under static conditions) indicated that the hydrodynamic shear forces were low and not detrimental to their structure with the hydrogel still supporting and shielding the cell spheroids from direct contact with shear stress. The theoretical values of the flow shear stress in the bioreactor fitted within the lower range of physiological shear stress (Chau et al., 2009).

Unlike most bioreactors currently developed for Tissue Engineering which are application-specific (Hirt et al., 2015; Tocchio et al., 2015; Raveling et al., 2018; Lim et al., 2019; Zohar et al., 2019), this system constitutes a multipurpose bioreactor for any study that requires cell encapsulation and perfusion conditions where is possible to select the cell type and customize the hydrogel accordingly. Its simple setup enables operation inside a CO_2_ incubator which controls the environmental parameters (temperature, pH, CO_2_), with the fluid flow rate and active gassing of the reservoir regulated externally by a peristaltic pump and a flow meter, respectively, and thus, allowing to adjusting the culture conditions to each cell type. Yet, the bioreactor could be integrated with an automatic control system for processes and studies that require tight monitoring of the culture conditions.

Other generic laboratory-scale systems such as the miniaturized stirred tank bioreactor could provide a dynamic environment and similar cellular yields but at the cost of greater hydrodynamic shear stress and turbulent regime due to mechanical agitation, consequently not replicating the physiological conditions and causing cell/scaffold damage (Cherry and Papoutsakis, 1986; Gareau et al., 2014; Santo et al., 2016). Alternatively, rotating wall vessels produce laminar flows with associated low hydrodynamic shear which suspend constructs in a microgravity environment, resultant from the cancelation of the centrifugal, drag and gravity force, but this mechanism is not identical to the *in vivo* perfusion. Another design not as widely used but that more closely mimics perfusion *in vitro* are the membrane bioreactors, particularly, hollow fiber bioreactors, where biomass and fluid are separated by a microporous membrane and the biomass grows in a 3D structure around the fibers. However, it is subjected to membrane fouling and formation of concentration gradients limiting mass transfer to cells further apart from the membrane. Compared to these systems, the sFBB sustains perfusion of several individual 3D cell micro-constructs at the expense of a hydrodynamic environment in laminar regime, diminishing the creation of mass transfer impairments and also benefiting from easier sampling and media exchange since cells are encapsulated in individual beads and confined to the main column.

While the current prototype stands as an obvious pilot scale for process optimization of the clinical FBB, the choice of scale for the sFBB concerned the capacity of the design to host enough biomass for multiple observations per sample and time points, as each bead constitutes a tissue-like structure, and in relevant volumes relatable to *in vivo* conditions and models (e.g., small animal liver). These operating volumes will not support high-throughput analysis such as microfluidics (Tumarkin et al., 2011). Micro-bioreactors, and those operating in the milliliter scale, have been suitable platforms for drug-screening, stem cell differentiation protocols or organ-on-a-chip devices as they enable expensive processes to be conducted in a cost-effective way, a consequence of the small operating volumes, otherwise prohibitive at larger scales (Kane et al., 2019; Rajan et al., 2020; Zhao et al., 2020). However, the sFBB system offers an appropriate scale, operating in perfusion mode, for the intermediate stage of testing methods in a more physiologically relevant volume or expanding optimized protocols. It widens the variety of platforms to be used in the R&D process, providing advantageous *in vitro* alternatives and potentially minimizing the number of animals needed in research.

Moreover, the system robustness, reproducibility and flexibility were demonstrated through expansion into a parallel multiunit setup of up to 4 sFBBs, producing equivalent final cell densities and fluidization levels. Of note, differences in fluidization level among bioreactors, specially until day 4, are a result of setup inherent variability and beads adjusting to the dynamic environment. These characteristics are similar to other parallelised small-scale technologies (Tai et al., 2015; Qian et al., 2016; Lovecchio et al., 2019; Valls-Margarit et al., 2019), and their validation demonstrates that the prototype could enable simultaneous multiple condition observations and/or establishment of co-culture protocols. It improves the throughput character of the sFBB, as well as, the diverse range of applications from cellular expansion to investigation of cellular responses to external agents and stresses (e.g., hydrodynamic forces, differentiation factors), and paracrine effects of different cells types under co-culture, for example.

## Conclusion

The sFBB demonstrated the feasibility of scaling down FBB technology to a simple, one-piece design, sustaining the same biological performance with the additional advantage of operating at smaller volumes and parallel multiunit setups. This prototype could serve as a device for long-term culture and analysis of 3D cell constructs with prospects of engineering *in vitro* models, which could more closely reproduce the *in vivo* conditions and in a more cost-effective manner, using physiological comparable volumes, and ultimately contribute toward reducing the use of animal models in research.

## Data Availability Statement

All datasets generated for this study are included in the article/Supplementary Material.

## Author Contributions

JM conceived the study, designed the experiments, and wrote the manuscript. JM, EE, MA, and S-AC performed the experimental work. JM ran the computational models. BF and CS were involved in the experimental design. All authors contributed to manuscript revision, read and approved the submitted version.

## Conflict of Interest

The authors declare that the research was conducted in the absence of any commercial or financial relationships that could be construed as a potential conflict of interest.
