# Supplementary material for: Small-Scale Fluidized Bed Bioreactor for Long-Term Dynamic Culture of 3D Cell Constructs and in vitro Testing
Source: Front Bioeng Biotechnol. 2020 Aug 20;8:895. doi: 10.3389/fbioe.2020.00895 (PMC7468403; doi:10.3389/fbioe.2020.00895)
Supplement: Supplementary file 1 [file Data_Sheet_1.docx]

Supplementary Material

**
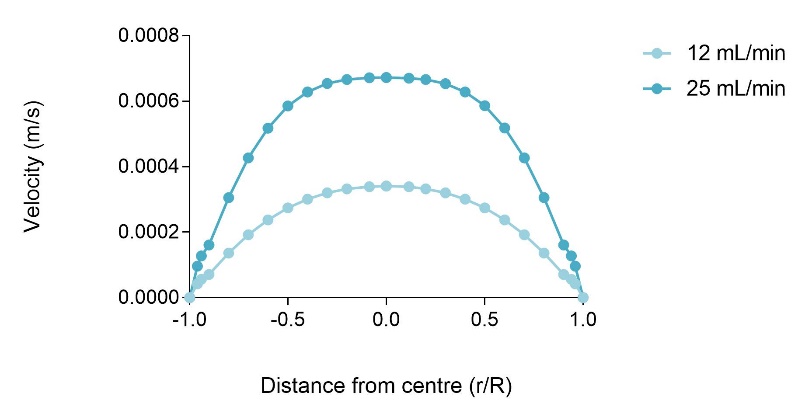
**

**Supplementary Figure 1.** Velocity profile of fluid inside small-scale fluidised bed bioreactor (sFBB). Velocity of the fluid in the sFBB at inlet flow rates of 12 and 25 mL/min measured at the top plane 0.062 m from the base of the bioreactor. Values presented in m/s.

Supplementary Table 1. Table 4 – Theoretical prediction of minimum fluidization velocity (u_mf_). umf calculated using Ergun, Kozeny-Carman, Wen and Yu, and Riba models. d – diameter; ρ – density; μ – viscosity; ε – settled bed porosity; φ – alginate encapsulated cells shape factor.

| **Minimum fluidisation velocity parameters** | | | |
| --- | --- | --- | --- |
| **d_bead_ (μm)** | 596 | **ρ_fluid_ (g/mL)** | 1.005 |
| **ρ_bead_ (g/mL)** | 1.04 | **μ_fluid_ (cP)** | 0.94 |
| **ε** | 0.42 | **Gravitational constant (m/s^2^)** | 9.81 |
| **φ** | 0.96 |  |  |
| **Predicted minimum fluidisation velocity (m/s)** | | | |
| **Ergun** | 9.81x10^-5^ | | |
| **Konzeny-Carman** | 8.89x10^-5^ | | |
| **Wen and Yu** | 7.80x10^-5^ | | |
| **Riba** | 3.83x10^-4^ | | |
